# Intertemporal preference reversals are associated with early activation of insula and sustained preferential processing of immediate rewards in visual cortex

**DOI:** 10.1038/s41598-021-01579-7

**Published:** 2021-11-15

**Authors:** Sathya Narayana Sharma, Azizuddin Khan

**Affiliations:** Psychophysiology Laboratory, Department of Humanities and Social Sciences, Indian Institute of Technology Bombay, Mumbai, 400076 India

**Keywords:** Human behaviour, Decision

## Abstract

Decision makers tend to give magnified significance to immediately available rewards which leads to intertemporal preference reversals, which is a form of self-control failure. The objective of the present study was to understand the cognitive and neural underpinnings of this phenomenon using event-related potentials (ERP) and their source localization using standardized low-resolution brain electromagnetic tomography analysis (sLORETA). Twenty-four participants performed a money choice task, where they made choices between a smaller-sooner and a larger-later reward, which included trials with and without an immediately available option, while their electroencephalography (EEG) activity was recorded. Trials with and without immediacy were identical except that the latter involved a front-end delay added to both the rewards. Results showed that presence of immediacy made the choices significantly more impulsive. Presence of immediate reward elicited larger visual P2 and late positive potential (LPP), indicating enhanced capture of automatic and sustained attention respectively, and smaller N2, indicative of diminished engagement of cognitive control processes. Source localization revealed increased activity in the visual cortex in the presence of immediacy, signifying higher valuation. Higher activation of areas of insula during P2—suggesting increased awareness of visceral signals—predicted larger impulsive preference reversals. The results suggest that presence of immediate reward biases the choice very early during the decision making process by precipitating visceral states that triggers approach behaviour, and highlight the need to adopt strategies like precommitment to counter the effect.

## Introduction

Building blocks of a good life are sensible decisions. Many crucial choices that one needs to make during the course of one’s life are intertemporal in nature. These are typically choices between an outcome of lower value which is realized earlier in time and an outcome of higher value which is realized only later in time. For instance, imagine one having to decide between eating an extra piece of a dessert or sticking to one’s diet plan. Here, the health benefit derived from sticking to the diet plan, while being clearly of a higher value, is noticeable only over a longer time frame. Whereas, the pleasure of the dessert is immediately available. It is easy to notice that how one handles such scenarios is going to be of considerable consequence.

Immediacy effect refers to the tendency of decision makers to magnify the significance of immediately available rewards. This constitutes a violation of the stationarity axiom of the discounted utility model^1,2^, which posits that the preference between two rewards A and B depends only on the difference in the delays that they can be realised (apart from difference in the reward magnitudes), and therefore adding/subtracting the same delay to/from both the rewards does not affect the preference. For example, in order for stationarity to hold, if an individual prefers Rs. 500 today over Rs. 510 in 1 week, then they should also prefer Rs. 500 in a year over Rs. 510 in a year and 1 week, and vice versa. Not being consistent in this manner results in a preference reversal. The reversal in preference brought about by adding/subtracting a common delay to/from both the options is sometimes referred to as static or synchronic preference reversal^3,4^. Here, both the choices are made on the same occasion. For example, an individual may enthusiastically sign up for an exercise regimen that is supposed to begin next month at a gym, but may change the decision if informed it in fact begins today. Whereas in dynamic or diachronic preference reversal, the common delay decrement is allowed to elapse in actuality and the choices are made on two occasions. For example, an individual may have made a decision to quit smoking after a visit to a physician; but after a week when they are offered a cigarette by a co-worker, a preference reversal occurs and they decide to smoke “just this one”. It is important to note that static preference reversal necessarily implies dynamic preference reversal only under the assumption of time invariance, that is preferences do not depend on calendar time but only on relative temporal distances to the outcomes^5,6^.

Preference reversal caused by immediacy effect can be conceptualized as a mechanism for failure of self-control^7^. For example, individuals may state that they value their health more and want to eat healthy, but often succumb to the temptation at the last minute when an opportunity presents itself, that is, when a piece of dessert is sitting in front of them and they need to make a choice. Such apparent reversal of preference has also been argued to underlie drug dependence^8^.

Immediacy effect and preference reversal cannot be accounted for under the assumption that the value of future outcomes is discounted with respect to time at constant rate, such as exponential discounting^9^. Therefore, it has been suggested that future rewards are discounted hyperbolically^10^, whereby discount rates over shorter time horizons are larger than discount rates over longer time horizons. There has been substantial empirical evidence that shows that individuals’ discounting pattern is indeed better described by a hyperbolic function than an exponential function^4,11,12^. Hyperbolic discounting allows for preference reversal to occur; however, it is a descriptive model and the underlying factors that give rise to immediacy effect and preference reversal are not explained by it. Despite the centrality of preference reversal as an explanatory mechanism for various forms of self-control failure, direct empirical demonstrations of static and dynamic preference reversal are equivocal, and its underlying neural and electrophysiological correlates remain poorly understood.

Testing dynamic preference reversal in lab is costlier as it requires the participants to come to the lab for two sessions, separated from each other by possibly months. However, a few studies have attempted it, with mixed findings^5,6,13–15^. Among the studies that have attempted to test static preference reversals behaviourally, Keren and Roelofsma^16^ employed a between-subjects design with one choice problem and showed that only 37% of participants chose the earlier option when it was 26 weeks away, while 82% chose it when it could be obtained immediately. They argued that this occurred due to immediately available outcomes being perceived as more certain than delayed outcomes by individuals. Supporting this, they also showed that adding uncertainty to both the rewards greatly reduced such immediacy effect. Ainslie and Haendel^13^, Green et al.^17^, and Kirby and Herrnstein^18^ too found supporting evidence for static preference reversals. But, as pointed out by Kable^7^, these studies used an experimental procedure which could only detect preference reversals in the predicted direction, that is towards the larger-later option, as a common delay is introduced to both the rewards. Further, Bleichrodt and Johannesson^19^ demonstrated violation of stationarity while choosing between health outcomes, which they argued might have stemmed from immediacy effect. Consistent with the idea that preference reversal may underlie addiction, Pope et al.^20^ showed that the common delay increment that was required to elicit a static reversal of preference from smaller-sooner reward to larger-later reward was more for smokers than non-smokers. Whereas, other studies^21–23^ have failed to find evidence for violations of stationarity or preference reversals.

There have been a few attempts to study the neural bases of immediacy effect as well. McClure et al.^24^ showed that limbic structures including ventral striatum (VS), posterior cingulate cortex (PCC), and medial prefrontal cortex (mPFC) were preferentially activated when participants were considering choices that involved an immediate outcome. Whereas, regions of the lateral prefrontal cortex and posterior parietal cortex were activated for all types of intertemporal choices. They invoked a two-systems model and argued that the limbic structures represented an impatient system and the lateral prefrontal and posterior parietal cortical structures represented a patient system, whose competition governs the choice behaviour. However, this account was challenged by the results obtained by Kable and Glimcher^25,26^ who showed that neural activity in VS, PCC, and mPFC tracked subjective values of both immediate and delayed rewards. They however did not find evidence for preference reversals in their dataset, which they explained by arguing that the hyperbolicity of the discounting of a delayed reward is not anchored to the immediate present, but to the time of the soonest possible reward. Sripada et al.^27^ found that while VS, mPFC, and PCC reflected the difference in subjective value of the later minus the earlier reward, mPFC and PCC also reflected the presence of an immediate reward in the choice options. However, they also did not find behavioural evidence for immediacy effect in their data set, which they argued might have been because of the relatively low reward magnitudes and narrow range of delays used. Further, Sellitto et al.^28^ showed that patients who had damage to the insular cortex were less sensitive to sooner rewards than healthy individuals. Crucially, they also demonstrated that insular patients were relatively less aroused by positively valenced stimuli, strongly suggesting that insular damage likely dampened the incentive salience of sooner rewards by impairing somatic signals that indicate their desirability.

We argue that it may be problematic to use the same set of choice problems across participants, as the experienced difficulty while responding to the trials will vary across participants and may become a confound, which may partly explain the lack of behavioural evidence for immediacy effect in some of the studies^27^ that attempted to identify its neural components. In the present study, we measured the discount rates of individuals initially and used them to construct choice problems, thereby keeping the experienced difficulty identical across participants. Further, although it has been hinted^27,29^ that the reward magnitude and the delays used may influence preference reversal, there has been little effort in systematically varying these two attributes to observe their effects, which also may partly account for the equivocal findings in the literature. We addressed this concern too in the present study. Also, we used random presentation of trials which makes the method capable to detect preference reversals in either direction, unlike in some previous studies^17,18^ which have used a method that explicitly probes for preference reversal only towards the predicted direction, that is from impulsive choice to patient choice as a common delay is introduced to both the reward options. We also tested the relationship between degree of preference reversal and trait impulsivity, which is defined as a stable personality characteristic^30^. Although Sripada et al.^27^ attempted to assess this, their data set did not show occurrence of preference reversal. The present study, using a more sensitive and difficulty-matched choice task, hoped to test this association.

Along with systematically investigating the behavioural effects of immediacy using a static preference reversal paradigm, the present study also attempted to examine the cognitive and neural processes underlying immediacy and preference reversal. Towards this purpose we used event-related potentials (ERPs) and their source localisation. While neuroimaging is helpful in identifying the anatomical structures involved in executing a behaviour, ERPs are especially useful in zeroing in on specific cognitive processes underlying a behaviour. Electroencephalography (EEG) signal recorded from the scalp contains several ERPs embedded within it, which are associated with specific cognitive events, and can be extracted by averaging the signal across many trials and then grand-averaging across many participants^31^. Source localisation of ERPs using standardized low-resolution brain electromagnetic tomography analysis (sLORETA) was also conducted to obtain an estimation of the cortical sources whose activity was related to immediacy effect and preference reversal.

Previous studies conducted on intertemporal choice have revealed a variety of ERP components that are elicited during decision making process. The positive-polarity stimulus-locked P2 has been shown to reflect an initial evaluation of the reward and time delay^32,33^. P2 has also been shown to be higher for individuals preferring immediate reward more often^34^, and for individuals high on procrastination^35^. P2 has been understood to be an attention-related component which has been shown to be modulated by affective valence of the stimulus^36–38^.

The negative-polarity N2 has been found to be smaller for trials that resulted in impulsive choices than patient choices^32^. N2 has also been shown to be smaller for individuals preferring immediate reward more often^34^. N2 has been argued to be sensitive to response inhibition and cognitive control^39^. Populations such as individuals who smoke, who tend to have increased intertemporal impulsivity^40^, have been also shown to have diminished no-go N2 in a go/no-go task^41^, reflecting diminished cognitive control.

Finally, the positive-polarity late-latency components P3 and Late Positive Potential (LPP) have also been shown to be elicited during intertemporal decision making^32,34^. Both these components, sometimes combined as P3/LPP complex^42^, have been argued to reflect allocation of sustained attention to and elaborative processing of motivationally salient stimuli^43^.

We predicted that immediacy would elicit more impulsive choices behaviourally and elicit enhanced attentional response during the initial detection phase, which would be reflected as a larger P2. We also hypothesized that presence of immediate reward would cause a smaller N2 component indicating diminished cognitive control processes. We predicted that immediacy would trigger the appetitive motivational systems in the brain and capture more sustained attentional resources which would be reflected as larger P3/LPP. Further, source localisation of ERP components elicited by intertemporal choice tasks has been rare. Therefore, to understand the neural correlates of immediacy and preference reversal, an attempt was made to localize the cortical sources whose activity during the time course of the ERPs significantly differed between conditions, and also whose differential activation predicted the degree of preference reversal.

## Results

### Behavioural data

The mean percentage of impulsive choices (choosing smaller-sooner reward) was calculated under Immediacy Present (IP) and Immediacy Absent (IA) conditions and the descriptive statistics are shown in Table 1. A repeated-measures analysis of variance (ANOVA) with Immediacy (IP, IA), Reward Magnitude (Small, Large), and Delay Magnitude (Small, Large) as within-subjects factors and percentage of impulsive choices as the dependent variable showed that the main effect of Immediacy was significant (*F*(1, 23) = 32.77, *η*_*p*_^2^ = 0.59, Cohen’s *f* = 1.20, *p* < 0.001, observed power > 0.99). While under IP condition the number of impulsive choices were 61.43%, under IA condition, it came down to 28.49%. The main effect of Reward Magnitude (*F*(1, 23) = 0.12, *η*_*p*_^2^ = 0.005, Cohen’s *f* = 0.07, *p* = 0.73, observed power = 0.06) and of Delay Magnitude (*F*(1, 23) = 1.03, *η*_*p*_^2^ = 0.04, Cohen’s *f* = 0.20, *p* = 0.32, observed power = 0.16) were not statistically significant, neither were any interactions.

**Table 1:** Mean values and standard deviations (in parenthesis) of percentage of impulsive choices under all experimental conditions.

Delay magnitude	Immediacy present		Immediacy absent	
	Reward magnitude		Reward magnitude	
	Small	Large	Small	Large
Small	64.48 (14.14)	61.77 (23.87)	29.48 (27.66)	28.23 (23.38)
Large	60.52 (17.15)	58.96 (24.36)	28.12 (25.46)	28.12 (22.30)
	61.43 (20.13)		28.49 (24.40)	

### Event related potentials

**Figure 1:**
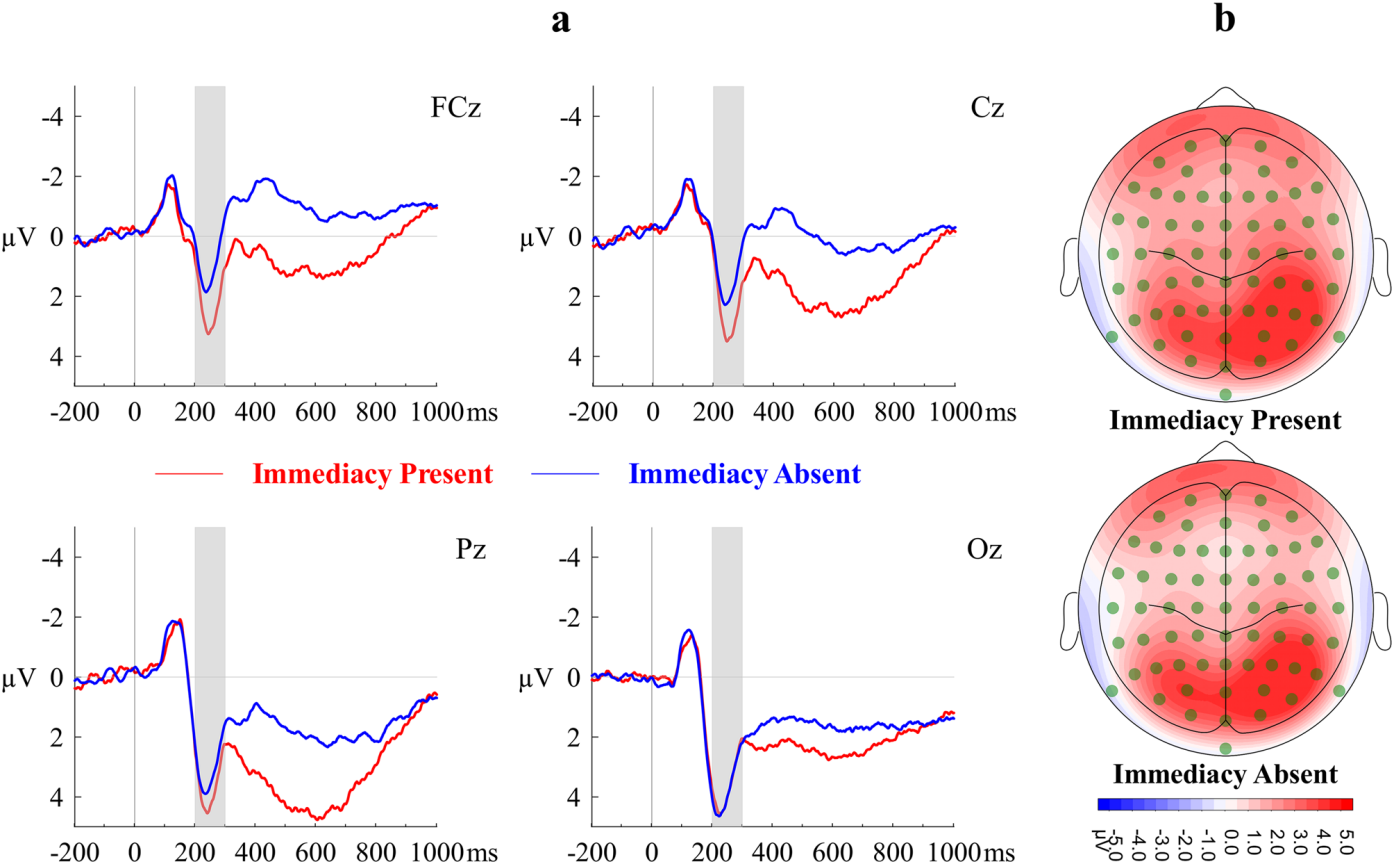
(**a**) Grand averaged stimulus-locked ERP waveforms of P2 component at representative electrodes. (**b**) Topographical maps of mean surface potential during P2 time window. Immediacy Present (IP) and Immediacy Absent (IA) conditions are contrasted.

A repeated-measures ANOVA with Immediacy (IP, IA) and Region (Frontocentral, Parietal, Occipital) as within-subjects factors and mean amplitude of P2 as the dependent variable showed that the main effect of Immediacy (*F*(1, 23) = 6.24, *η*_*p*_^2^ = 0.21, Cohen’s *f* = 0.52, *p* = 0.02, observed power = 0.67), and of Region (*F*(1.16, 26.60) = 9.82, *η*_*p*_^2^ = 0.30, Cohen’s *f* = 0.65, *p* = 0.003, observed power = 0.89) were significant and so was their interaction (*F*(1.41, 32.48) = 22.54, *η*_*p*_^2^ = 0.50, Cohen’s *f* = 1.00, *p* < 0.001, observed power > 0.99). Overall, P2 component was significantly larger under IP condition (*M* = 4.00, *SE* = 0.50) than under IA condition (*M* = 3.49, *SE* = 0.55). Post-hoc Bonferroni-corrected pairwise comparisons revealed that the effect of Immediacy was largest in the frontocentral region (*M*_IP_ = 2.64, *SE*_IP_ = 0.66; *M*_IA_ = 1.63, *SE*_IA_ = 0.67, Cohen’s *d*_*z*_ = 0.84, *p* < 0.001), smaller but significant (*Mean difference* = 0.62, Cohen’s *d*_*z*_ = 0.49, *p* = 0.02) in the parietal region, and non-significant (*Mean difference*  = − 0.12, Cohen’s *d*_*z*_  = − 0.16, *p* = 0.42) in the occipital region. Figure 1 shows the waveform of P2 and the corresponding scalp topography.

**Figure 2:**
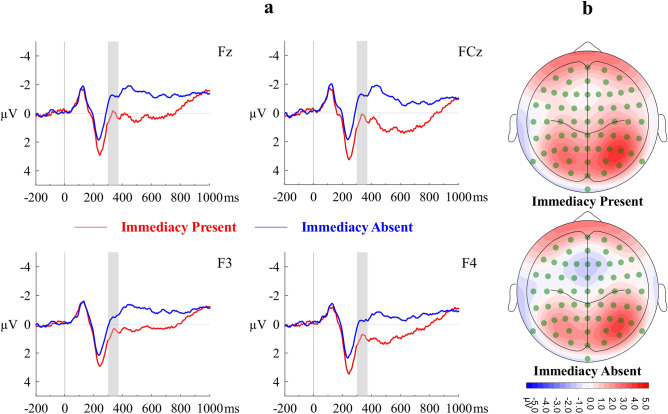
(**a**) Grand averaged stimulus-locked ERP waveforms of N2 component at representative electrodes. (**b**) Topographical maps of mean surface potential during N2 time window. Immediacy Present (IP) and Immediacy Absent (IA) conditions are contrasted.

A repeated-measures ANOVA with Immediacy (IP, IA) and Electrode Position (Fz, F3, F4, FCz, FC3, FC4) as within-subjects factors and mean amplitude of N2 as the dependent variable showed that the main effects of Immediacy (*F*(1, 23) = 12.76, *η*_*p*_^2^ = 0.36, Cohen’s *f* = 0.75, *p* = 0.002, observed power = 0.93) and of Electrode Position (*F*(3.28, 75.44) = 10.70, *η*_*p*_^2^ = 0.32, Cohen’s *f* = 0.68, *p* < 0.001, observed power > 0.99) were statistically significant, but not their interaction (*F*(3.40, 78.37) = 2.52, *η*_*p*_^2^ = 0.01, Cohen’s *f* = 0.10, *p* = 0.06, observed power = 0.64). The N2 component had a significantly larger negativity under IA condition (*M* = − 0.49, *SE* = 0.66) than under IP condition (*M* = 0.73, *SE* = 0.68). Figure 2 shows the waveform of N2 and the corresponding scalp topography.

**Figure 3:**
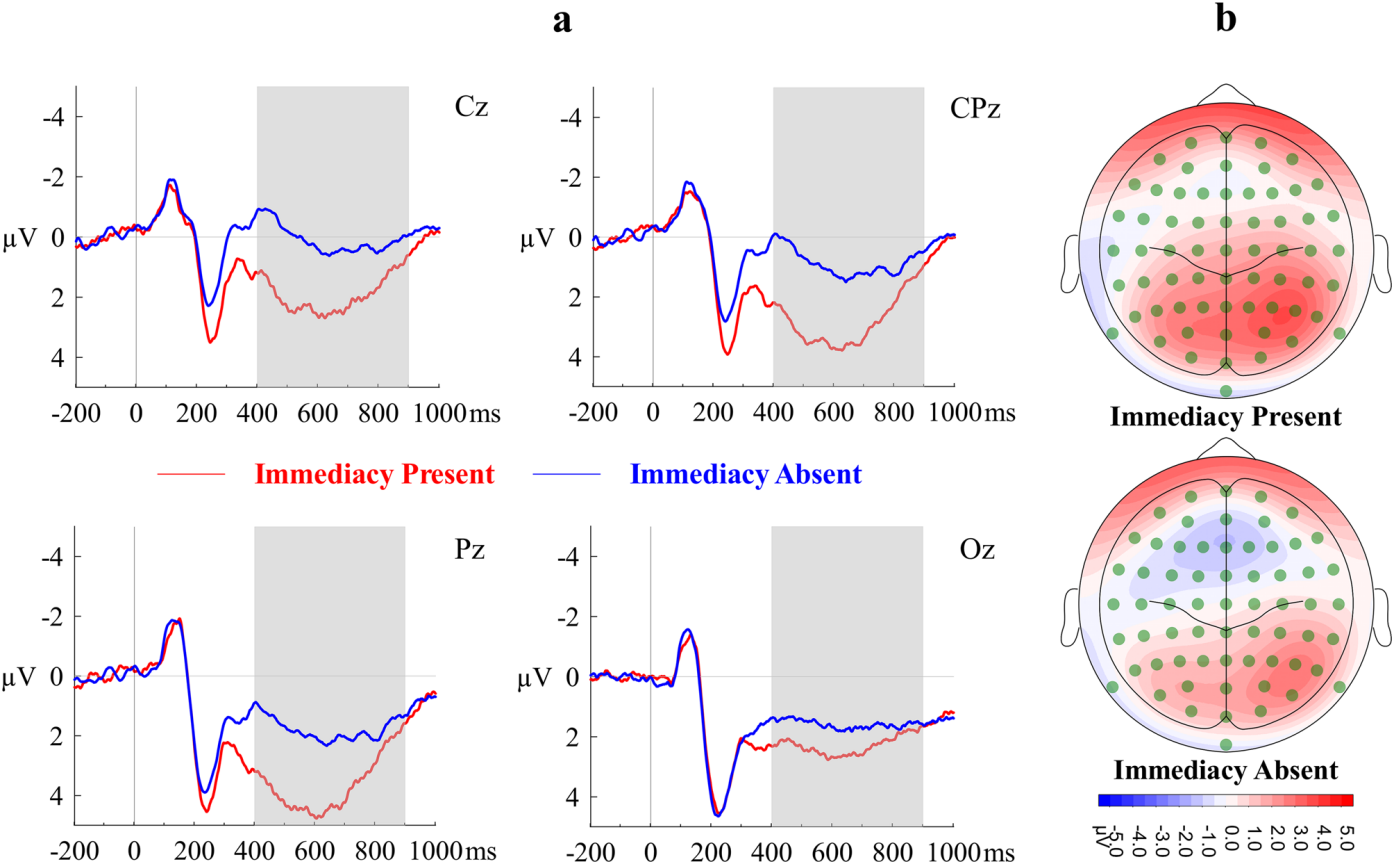
(**a**) Grand averaged stimulus-locked ERP waveforms of LPP component at representative electrodes. (**b**) Topographical maps of mean surface potential during LPP time window. Immediacy Present (IP) and Immediacy Absent (IA) conditions are contrasted.

A repeated-measures ANOVA with Immediacy (IP, IA) and Region (Centroparietal, Occipital) as within-subjects factors and mean amplitude of LPP as the dependent variable showed that the main effect of Immediacy was significant (*F*(1, 23) = 24.98, *η*_*p*_^2^ = 0.52, Cohen’s *f* = 1.04, *p* < 0.001, observed power > 0.99), whereas the main effect of Region (*F*(1, 23) = 1.42, *η*_*p*_^2^ = 0.06, Cohen’s *f* = 0.25, *p* = 0.24, observed power = 0.21) was not. The interaction effect was significant (*F*(1, 23) = 13.93, *η*_*p*_^2^ = 0.38, Cohen’s *f* = 0.78, *p* = 0.001, observed power = 0.95). Overall, LPP component was significantly larger under IP condition (*M* = 2.93, *SE* = 0.50) than under IA condition (*M* = 1.54, *SE* = 0.46). Post-hoc Bonferroni-corrected pairwise comparisons revealed that the effect of immediacy was larger in the centroparietal region (*M*_IP_ = 2.76, *SE*_IP_ = 0.68; *M*_IA_ = 0.96, *SE*_IA_ = 0.59, Cohen’s *d*_*z*_ = 1.02, *p* < 0.001), and smaller but significant (*Mean difference* = 0.99, Cohen’s *d*_*z*_ = 0.89, *p* < 0.001) in the occipital region. Figure 3 shows the waveform of LPP and the corresponding scalp topography.

**Figure 4:**
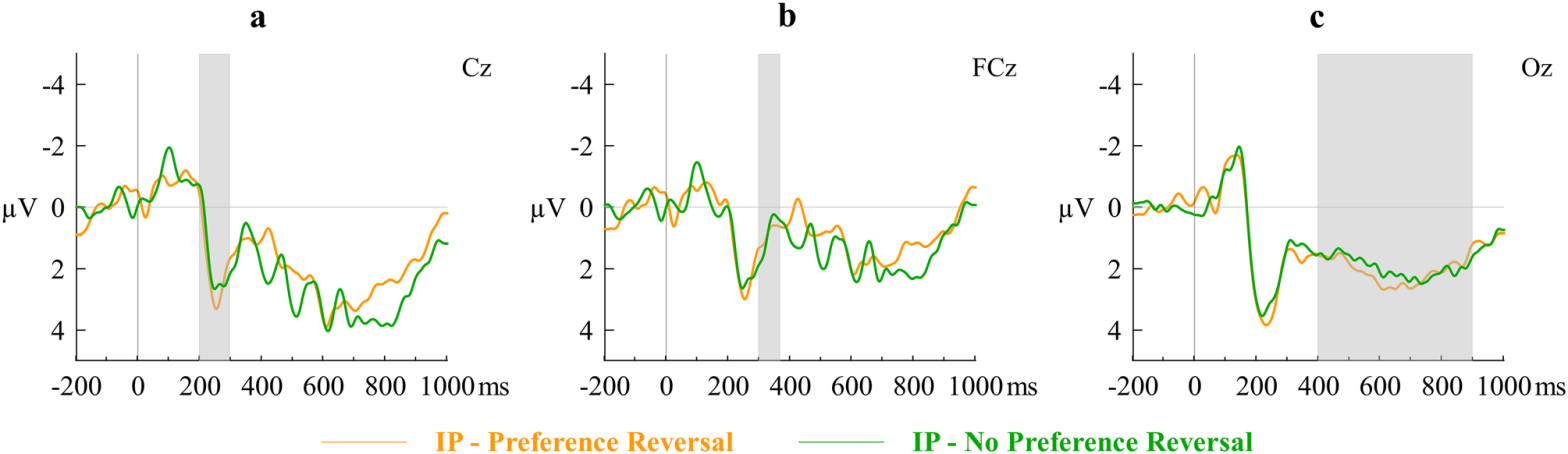
Grand averaged stimulus-locked ERP waveforms of (**a**) P2, (**b**) N2, and (**c**) LPP at representative electrodes. Immediacy Present trials associated with preference reversal (IP-PR) and not associated with preference reversal (IP-NPR) are contrasted.

Repeated-measures ANOVAs showed that mean amplitudes of P2 (*F*(1, 9) = 0.62, *η*_*p*_^2^ = 0.06, Cohen’s *f* = 0.25, *p* = 0.45, observed power = 0.11), N2 (*F*(1, 9) = 0.03, *η*_*p*_^2^ = 0.004, Cohen’s *f* = 0.06, *p* = 0.86, observed power = 0.05), and LPP (*F*(1, 9) = 0.292, *η*_*p*_^2^ = 0.03, Cohen’s *f* = 0.18, *p* = 0.60, observed power = 0.07) did not vary across the sets of IP trials—in which the immediate reward was chosen—associated with preference reversal (IP-PR) and with no preference reversal (IP-NPR). None of the condition x electrode site interactions were statistically significant. Figure 4 shows the waveforms at representative electrode sites.

**Figure 5:**
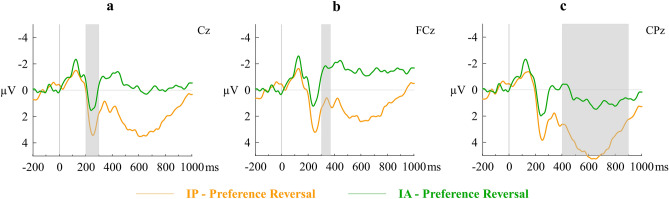
Grand averaged stimulus-locked ERP waveforms of (**a**) P2, (**b**) N2, and (**c**) LPP at representative electrodes. Immediacy Present and Immediacy Absent trials associated with preference reversal are contrasted (IP-PR vs IA-PR).

ERPs elicited by IP trials associated with preference reversal (IP-PR) and IA trials associated with preference reversal (IA-PR) were contrasted next. A repeated-measures ANOVA with Immediacy-PR (IP-PR, IA-PR) and Region as within subject factors and the mean amplitude of P2 as the dependent variable showed that the main effect of Immediacy-PR (*F*(1, 21) = 7.41, *η*_*p*_^2^ = 0.26, Cohen’s *f* = 0.59, *p* = 0.01, observed power = 0.74), Region (*F*(1.16, 24.28) = 9.54, *η*_*p*_^2^ = 0.31, Cohen’s *f* = 0.67, *p* = 0.004, observed power = 0.88), and their interaction (*F*(1.36, 28.57) = 18.38, *η*_*p*_^2^ = 0.47, Cohen’s *f* = 0.94, *p* < 0.001, observed power = 0.99) were significant. Post-hoc comparisons showed that difference in P2 across IP-PR and IA-PR was largest in the frontocentral region (*Mean difference* = 1.54, Cohen’s *d*_*z*_ = 0.89, *p* < 0.001). Similarly, the main effect of Immediacy-PR (*F*(1, 21) = 12.79, *η*_*p*_^2^ = 0.38, Cohen’s *f* = 0.78, *p* = 0.002, observed power = 0.93) and of Electrode Position (*F*(3.05, 64.09) = 8.24, *η*_*p*_^2^ = 0.28, Cohen’s *f* = 0.62, *p* =  < 0.001, observed power = 0.99) on N2 were significant, but not their interaction. Finally, the main effect of Immediacy-PR on LPP was significant (*F*(1, 21) = 25.54, *η*_*p*_^2^ = 0.55, Cohen’s *f* = 1.10, *p* < 0.001, observed power = 0.99), but not the main effect of Region (*F*(1, 21) = 2.26, *η*_*p*_^2^ = 0.10, Cohen’s *f* = 0.33, *p* = 0.15, observed power = . 30). Their interaction effect was significant (*F*(1, 21) = 13.61, *η*_*p*_^2^ = 0.39, Cohen’s *f* = 0.80, *p* = 0.001, observed power = 0.94). Post-hoc comparisons showed that the difference in LPP across IP-PR and IA-PR was larger in the centroparietal region (*Mean difference* = 2.89, Cohen’s *d*_*z*_ = 1.06, *p* < 0.001). Figure 5 shows the waveforms at representative electrode sites.

### Source localization

**Figure 6:**
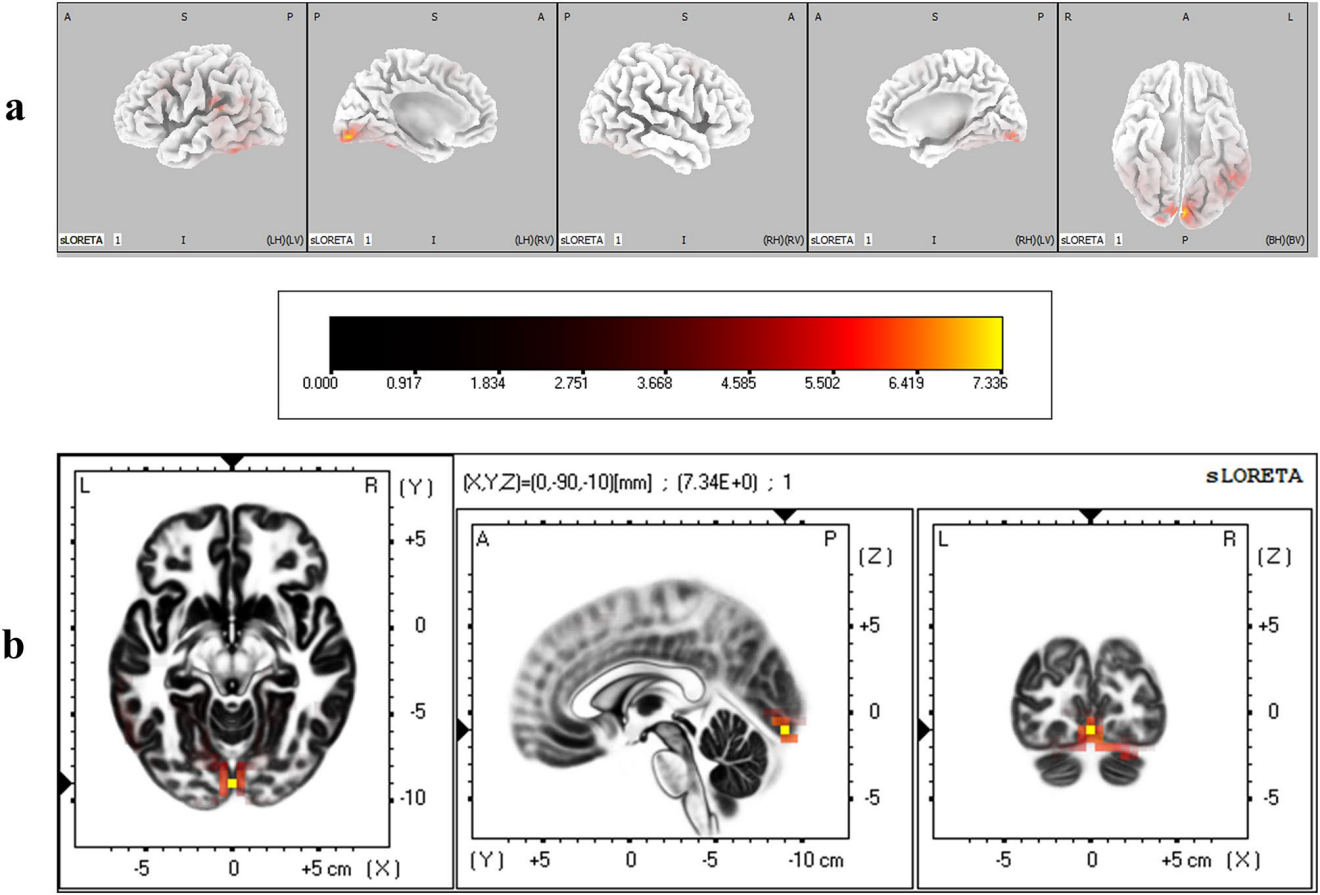
(**a**) Three dimensional sLORETA maps of cortical voxels that had significantly higher activation when immediacy was present (IP vs IA), during P2 time window. (**b**) Slice view of sLORETA source localization, focused on the MNI coordinate where maximum difference between conditions was revealed. The scale shows *t*-values such that larger values represent larger differences between conditions.

The cortical regions that were found to have significantly different degrees of activation between IP and IA conditions during the P2 time window are given in Table 2. It was found that lingual gyrus in occipital lobe, fusiform gyrus in both occipital and temporal lobes, superior temporal gyrus, and postcentral gyrus in parietal lobe were significantly more active when immediacy was present (corrected *p* < 0.05) (Fig. 6).

**Table 2:** Brain regions that showed significant differential activation between immediacy conditions during P2 time window.

Lobe	Structure	Brodmann area	MNI coordinates (x, y, z)	*t*-statistic
**IP > IA**				
Occipital	Lingual gyrus	17	− 5, − 90, − 10	6.15
		17	− 5, − 95, − 10	5.91
		17	5, − 90, − 10	6.09
		18	− 5, − 85, − 10	5.99
		18	− 5, − 85, − 5	5.77
		18	− 5, − 90, − 15	5.74
		18	0, − 90, − 10	7.34
		18	0, − 85, − 5	6.32
		18	0, − 90, − 15	6.25
		18	0, − 95, − 15	6.22
		18	0, − 90, − 5	6.15
		18	5, − 90, − 15	6.40
		18	5, − 85, − 15	6.19
		18	5, − 90, − 20	6.06
		18	5, − 85, − 10	6.03
		18	10, − 90, − 20	6.13
		18	15, − 90, − 20	5.81
	Fusiform gyrus	18	20, − 90, − 25	5.76
Temporal	Fusiform gyrus	37	− 50, − 55, − 25	6.02
		37	− 55, − 50, − 25	6.01
		37	− 45, − 55, − 25	5.92
		37	− 50, − 50, − 25	5.84
	Superior temporal gyrus	42	− 65, − 35, 20	6.04
Parietal	Postcentral gyrus	40	− 60, − 30, 20	5.99

Critical *t* value for corrected *p* < .05 for IP > IA = 5.72.

**Figure 7:**
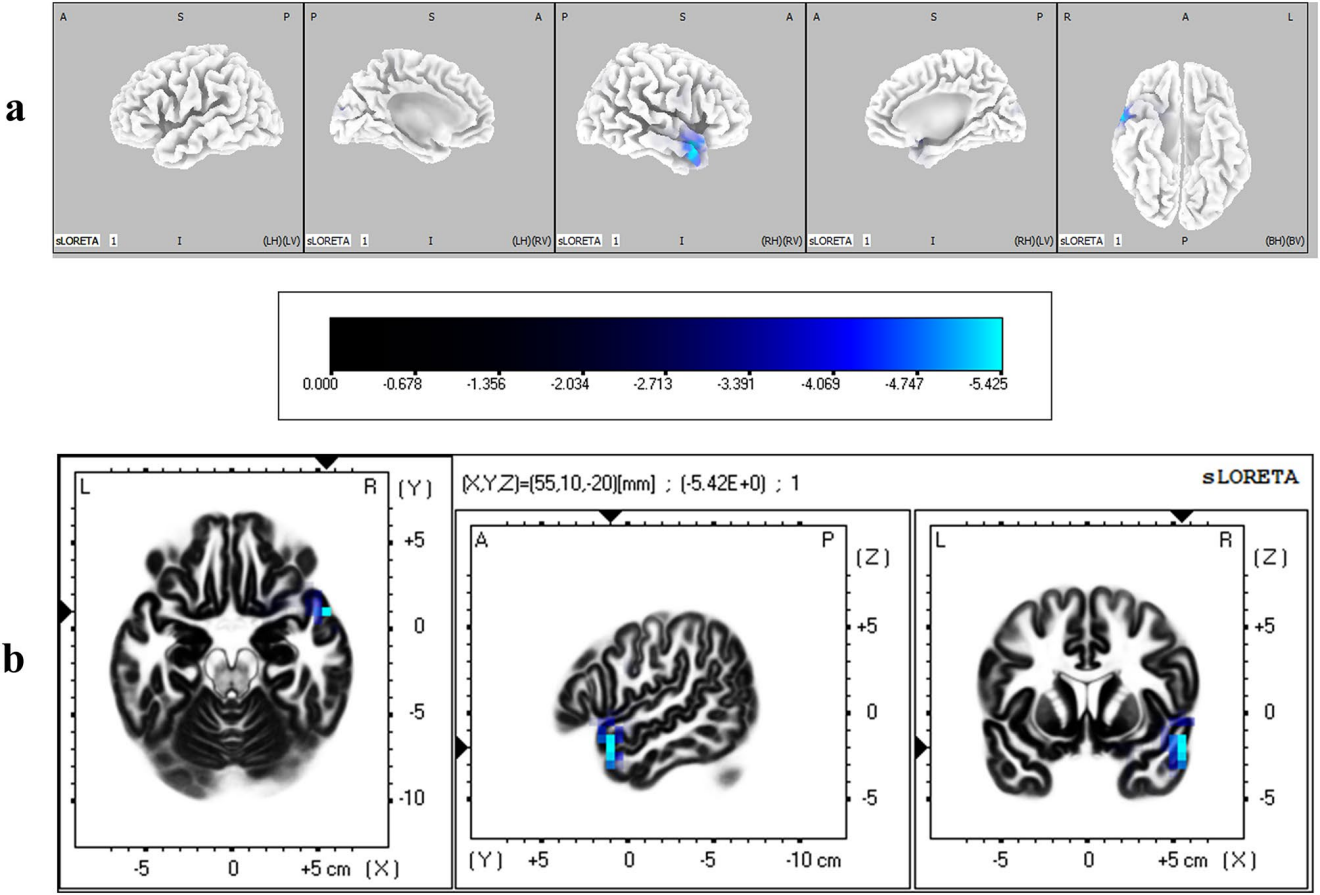
(**a**) Three dimensional sLORETA maps of cortical voxels that had significantly lower activation when immediacy was present (IP vs IA), during N2 time window. (**b**) Slice view of sLORETA source localization, focused on the MNI coordinate where maximum difference between conditions was revealed. The scale shows *t*-values such that smaller values represent larger differences between conditions.

**Figure 8:**
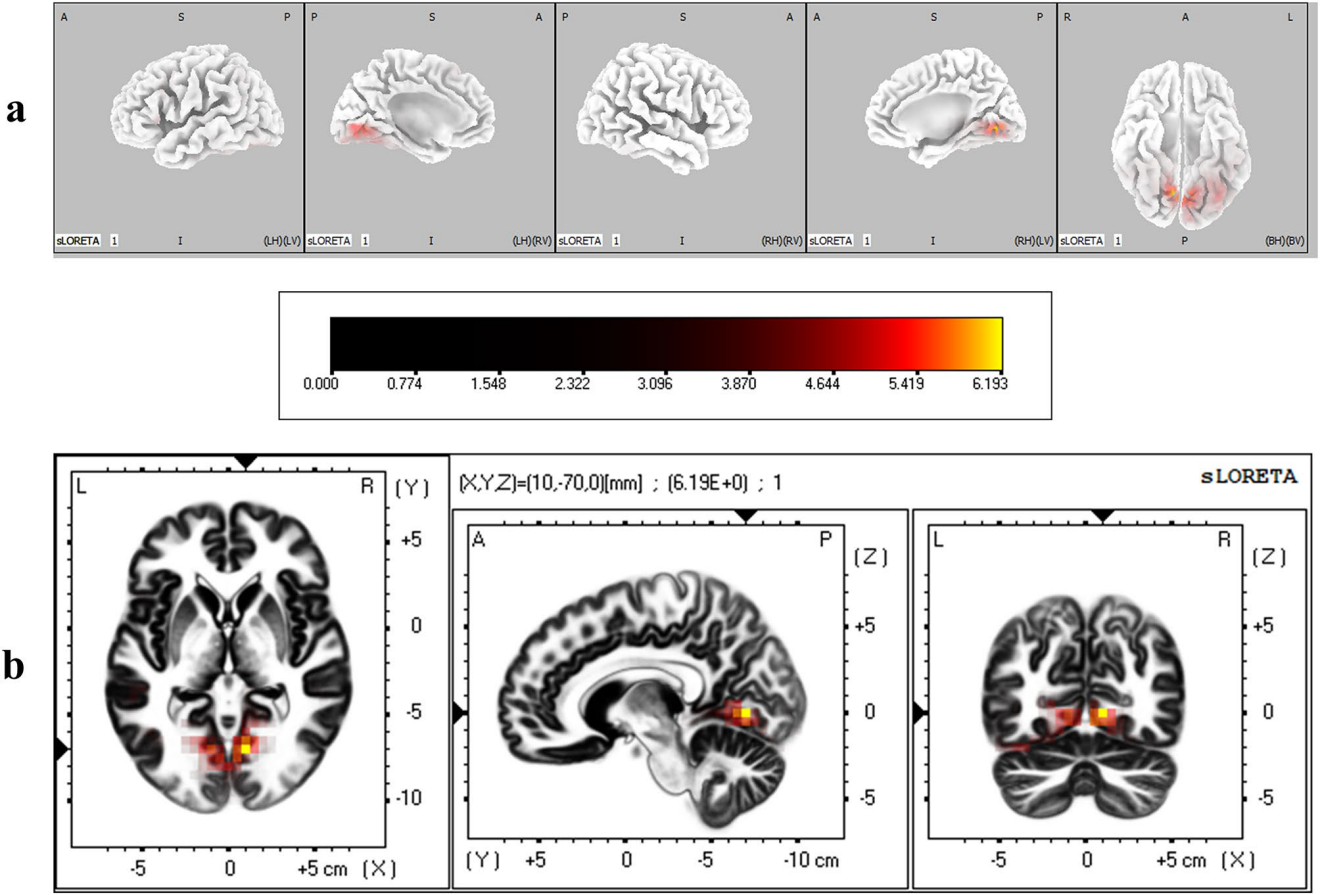
(**a**) Three dimensional sLORETA maps of cortical voxels that had significantly higher activation when immediacy was present (IP vs IA), during N2 time window. (**b**) Slice view of sLORETA source localization, focused on the MNI coordinate where maximum difference between conditions was revealed. The scale shows *t*-values such that larger values represent larger differences between conditions.

The cortical regions that were found to have significantly different degrees of activation between IP and IA conditions during the N2 time window are given in Table 3. It was found that middle temporal gyrus was significantly more active when immediacy was absent (corrected *p* < 0.05) (Fig. 7), and lingual gyrus in occipital lobe was significantly more active when immediacy was present (corrected *p* < 0.05) (Fig. 8).

**Table 3:** Brain regions that showed significant differential activation between immediacy conditions during N2 time window.

Lobe	Structure	Brodmann area	MNI coordinates (x, y, z)	*t*-statistic
**IP < IA**				
Temporal	Middle temporal gyrus	21	55, 10, − 20	− 5.42
**IP > IA**				
Occipital	Lingual gyrus	18	10, − 70, 0	6.19
		18	10, − 65, 0	5.79
		18	5, − 70, − 5	5.73
		18	5, − 70, 0	5.64
		18	10, − 70, − 5	5.63
		18	5, − 75, 0	5.57
		18	− 10, − 70, 0	5.56
		18	− 10, − 70, − 5	5.48
		18	15, − 70, − 5	5.46
		18	− 10, − 75, − 5	5.44
		19	15, − 65, 0	5.45

Critical *t* value for corrected *p* < 0.05 for IP < IA = − 5.42; Critical *t* value for corrected *p* < .05 for IP > IA = 5.44.

**Figure 9:**
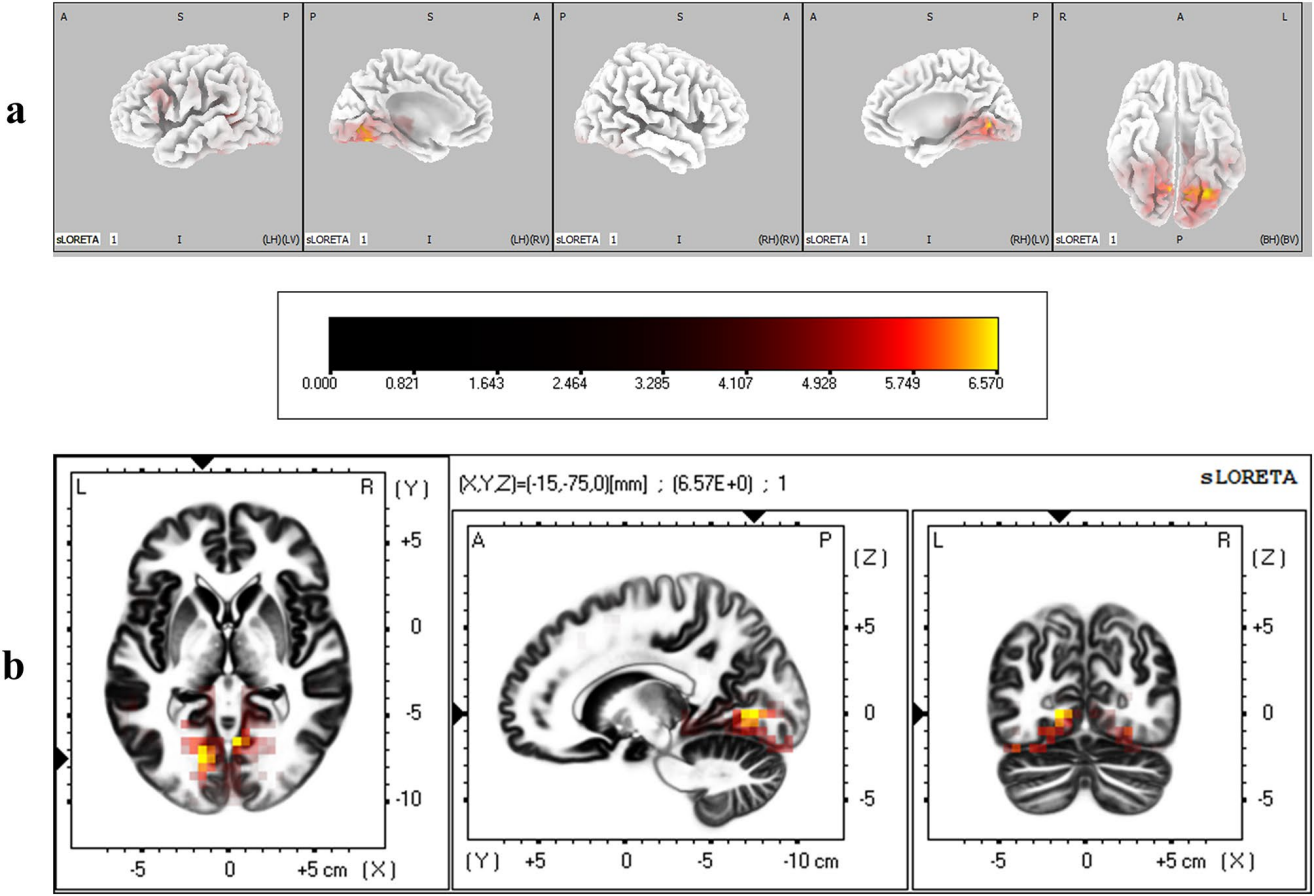
(**a**) Three dimensional sLORETA maps of cortical voxels that had significantly higher activation when immediacy was present (IP vs IA), during LPP time window. (**b**) Slice view of sLORETA source localization, focused on the MNI coordinate where maximum difference between conditions was revealed. The scale shows *t*-values such that larger values represent larger differences between conditions.

The cortical regions that were found to have significantly different degrees of activation between IP and IA conditions during the LPP time window are given in Table 4. It was found that lingual gyrus, fusiform gyrus, and cuneus in occipital lobe were significantly more active when immediacy was present (corrected *p* < 0.05) (Fig. 9).

**Table 4:** Brain regions that showed significant differential activation between immediacy conditions during LPP time window.

Lobe	Structure	Brodmann area	MNI coordinates (x, y, z)	*t*-statistic
**IP > IA**				
Occipital	Lingual gyrus	18	− 15, − 75, 0	6.57
		18	5, − 65, 0	6.54
		18	− 15, − 70, 0	6.49
		18	− 10, − 75, 0	6.29
		18	− 15, − 70, − 5	6.23
		18	− 15, − 75, − 5	6.20
		18	− 15, − 80, 0	6.18
		19	− 25, − 65, − 5	6.35
		19	− 20, − 70, − 5	6.21
		19	− 20, − 65, − 5	6.17
		19	− 20, − 70, − 10	6.13
	Fusiform gyrus	19	− 30, − 70, − 15	6.49
		19	− 25, − 70, − 15	6.20
		19	− 35, − 70, − 20	6.17
	Cuneus	30	5, − 65, 5	6.14

Critical *t* value for corrected *p* < 0.05 for IP > IA = 6.12.

Cortical activity elicited by IP-PR and IA-PR sets of trials were contrasted next. During P2 and N2, there were no statistically significant (corrected *p* < 0.05) differential activation. However, the areas whose activation differed the most between IP-PR and IA-PR was lingual gyrus (Brodmann Area, BA 18, IP > IA) during P2, and cuneus (BA 18, IP > IA) and middle temporal gyrus (BA 19, IP < IA) during N2. During LPP, cuneus (BA 23) and insula (BA13) had significantly (corrected *p* < 0.05) larger activation under IP-PR than IA-PR.

### Correlational results

**Figure 10:**
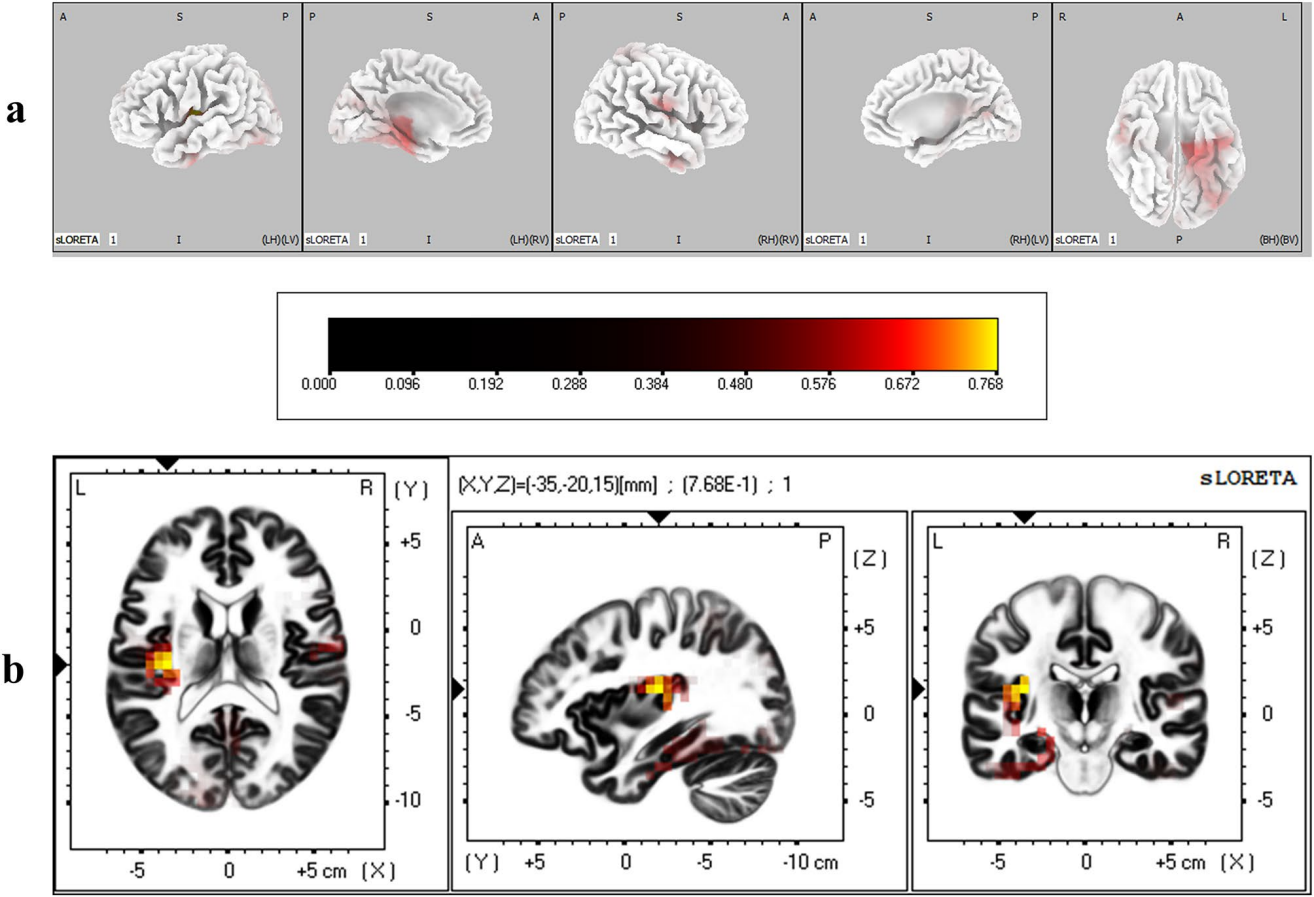
(**a**) Three dimensional sLORETA maps of cortical voxels whose differential activation between immediacy conditions (IP-IA) was significantly correlated with the degree of preference reversal, during P2 time window. (**b**) Slice view of sLORETA source localization, focused on the MNI coordinate whose differential activation had the highest correlation with the degree of preference reversal. The scale shows *r* values such that larger values represent larger positive correlations.

The degree of preference reversal was found to be not correlated with Barratt Impulsiveness Scale (BIS-11) total score (*r* = − 0.13, *p* = 0.53, observed power = 0.15). The differences in ERP amplitudes between immediacy conditions were not predictive of individual differences in the degree of preference reversal. Individual differences in the degree of preference reversal were found to be positively correlated with differential activation of insula between immediacy conditions (BA 13) during the P2 window (Table 5, Fig. 10). During N2 and LPP windows, there were no significant correlations.

**Table 5:** Brain regions whose differential activation between immediacy conditions significantly correlated with individual differences in degree of preference reversal.

Lobe	Structure	Brodmann area	MNI coordinates (x, y, z)	*r*	Slope
Sub-lobar	Insula	13	− 35, − 20, 15	0.77	0.02
		13	− 40, − 15, 10	0.76	0.02
		13	− 40, − 20, 15	0.76	0.02
		13	− 35, − 15, 15	0.75	0.02
		13	− 35, − 20, 20	0.75	0.02

Critical *r* value for corrected *p* < 0.05 for positive correlation = 0.75.

A summary of the results can be found in Table 6.

**Table 6:** Summary of the results.

	Findings
Behavioural	Percentage of impulsive choices changed from 61.43 when an immediate reward was present (immediacy present) to 28.49 when both rewards were delayed (immediacy absent)
ERP	Larger P2, LPP and smaller N2 in response to immediacy
Source Analysis	Increased activation of lingual, fusiform, superior temporal, and postcentral gyri during P2 in response to immediacy
	Increased activation of lingual gyrus and decreased activation of middle temporal gyrus during N2 in response to immediacy
	Increased activation of lingual and fusiform gyri and cuneus during LPP in response to immediacy
Correlational	During P2, differential activation of insula between the immediacy conditions predicted individual differences in degree of preference reversal

## Discussion

The present study aimed to demonstrate preference reversal phenomenon in intertemporal choice due to immediacy and investigate its electrophysiological and neural correlates. Three stimulus-locked event-related potentials were elicited by the choice problem, namely P2, N2, and LPP, all of which have previously been associated with intertemporal choice in the literature^32–34^. There was no discernible P3 that could reliably be differentiated from the larger LPP component. This is not surprising as P3 and LPP share many characteristics with each other including scalp distribution, latency, as well as functional significance^44^.

We measured the characteristic delay discount rate of individuals initially and used it to construct choice problems for the EEG task, thus ensuring that the experienced difficulty was identical across participants. The experiment involved hypothetical rather than real rewards, which is a common approach in intertemporal choice literature^45^, and various studies have reported no difference between delay discounting of real and hypothetical rewards^12,46^. The results showed occurrence of large preference reversals in the sample. The percentage of impulsive choices approximately halved as a common delay increment was added to both the options. While the mean percentage of impulsive choices was 61 when one option was immediate, it came down to 28 when both options were delayed in time, even as the time difference between the two outcomes was held constant. This pattern held for both small and large reward and delay magnitudes. Such static reversal of preference from smaller-sooner to larger-later reward as both the options are pushed farther in time is consistent with previous findings^13,16–18^. As our results showed that preference reversal was independent of reward and delay magnitude manipulations, the previous null results^27^ may not have been due to these factors, but may be attributable to the variable perceived difficulty levels across participants. Further, trait impulsivity as measured by BIS-11 was found to be not related to preference reversal. This is consistent with several studies that have found self-report measures of impulsivity to be not related with behavioural measures^47–49^, indicating that they tap into different facets of impulsiveness, which is not a unitary construct^30^.

The ERP analysis that contrasted trials with and without immediate reward showed that P2 and LPP components were larger, and N2 smaller while immediacy was present. P2 reflects an early attentional response, which orients the individual to execute rapid motor action^36^, and is enhanced for affectively valenced stimuli^50^. From an evolutionary perspective, a resource-scarce environment would select for quick attentional orientation and approach behaviour towards potentially rewarding stimuli which is immediately available. Consistent with this, we found larger P2 amplitudes when one of the options was an immediate reward. Littel and Franken^51^ have shown that a cue that was paired with smoking stimuli elicited larger P2 than one that was paired with neutral stimuli—only among smokers but not among non-smokers—indicating that P2 indeed reflected an enhanced early reflexive attention towards motivationally salient stimuli. Immediate reward can be argued to possess a motivational salience that is qualitatively different from and higher than delayed rewards, and which particularly captivates attention. These results are consistent with the earlier findings of P2 being higher for individuals preferring immediate reward more often^34^ and for those who are high on procrastinating behaviour^35^.

Results from source localization analysis showed that during the time course of P2, the activity in the primary visual cortex (BA 17 in lingual gyrus) and visual association areas (BA 18 in lingual and fusiform gyri) in the occipital lobe were higher when immediacy was present. Results from neuroimaging studies have demonstrated that motivationally salient and affectively valenced stimuli elicit significantly higher activity in the primary and secondary visual cortical areas^52–54^. Further, it has recently been shown that such higher activation does not merely indicate enhanced visual processing of affective stimuli, but that the visual system activity encodes affective content of such stimuli in a rich way^55^. Such higher activation of the visual association areas in the presence of immediacy continued during N2 (BAs 18 & 19 in lingual gyrus) and LPP (BAs 18 & 19 in lingual and fusiform gyri and BA 30 in cuneus) as well. Increased activation of the visual cortex in response to salient and arousing stimuli during decision making tasks has previously been reported, and has been argued to represent an adaptive mechanism to enhance the attentional processing of motivationally salient information^56,57^. Affect-related activation of visual cortex has also been argued to incorporate both bottom-up response to the stimulus and a top-down evaluation of it^55^, indicating that the higher activity induced by immediacy may represent a higher subjective valuation of the immediately available reward. The early visual cortical activation during P2 also spilled over to fusiform (BA 37) and superior temporal gyri (BA 42) in temporal lobe, both of which have been found to be associated with visual perception and attentional processes^58,59^.

Source localization results further revealed that the differential activation of insula (BA13) between immediacy conditions during P2 was significantly correlated with individual differences in degree of preference reversal, such that increased activity in insula when immediacy was present predicted more impulsive preference reversals. A number of previous studies have reported activation of insula during intertemporal decision making. Consistent with the present results, Li et al.^34^ found increased activity in insula during P2 among earthquake survivors who also were more impulsive in an intertemporal choice task, which the authors interpreted as evidence for more emotional and intuitive decision making. Also, Luo et al.^60^ reported enhanced activation in insula in response to immediate rewards relative to preference-matched delayed rewards outside of a decision context, which they interpreted as evidence for an incentive bias. Demonstrating the crucial role of insula in intertemporal choice, Sellitto et al.^28^ showed that patients with insular damage displayed less delay discounting and were less sensitive to sooner rewards. In a control task, they also showed that insular patients were less aroused by positively valenced stimuli, which allowed them to conclude that insula may play a role in upregulating the incentive value of sooner rewards by signalling a visceral urge to obtain a reward as soon as possible. According to a model Sellitto et al*.* propose, insula facilitates emotionally biased decisions by relaying interoceptive signals to ventromedial prefrontal cortex and the ventral striatum. Activation of insula has previously been shown to be related to the awareness of visceral states of the body, indicating that it may represent subjective feeling states^61^. Insula has also been argued to serve a catalytic function for the impulsive system in the brain and in translating visceral states of the body into subjective experience of craving^62^. Consistent with this, this region has been implicated in addiction and has been proposed to represent the interoceptive effects of drug usage while also making this information available to consciousness and memory^63,64^. The increased activation of insula in the presence of immediacy during P2, which predicted more impulsive preference reversals, may therefore reflect an increased awareness of visceral signals that manifested as a “gut-feeling” that triggered approach behaviour, which biased the decision towards selecting immediate reward. Postcentral gyrus has also been shown to be associated with interoceptive awareness^65^, which also was significantly more active when immediacy was present in the current study, which however did not predict the choice behaviour as insular activation did.

ERP results also showed that N2 component was significantly more negative when both the options were delayed. N2 has been associated with various dimensions of cognitive control such as response inhibition, response conflict, and performance monitoring^39^. Presence of an immediate reward has been shown to impair the ability to inhibit a prepotent response^66^. Further, motivationally salient cues have been shown to elicit reduced N2 reflecting impaired inhibition^67^. The motivationally salient immediate reward was attentionally prioritized in the P2 stage, which we argue left less resources for cognitive control processes^68^. Further, the lack of early visceral signals during trials when immediacy was absent imposed greater response conflict during decision making. Both these factors contributed to a larger N2 component when both the options were delayed. This is consistent with the previous results where larger N2 has been associated with delayed rewards^69^. Further, source localization results showed middle temporal gyrus to be significantly more active during N2 when both the options were delayed. Middle temporal gyrus, which has been shown to be functionally connected with frontal regions^70^, has previously been associated with cognitive control^71,72^.

Further, LPP which reflects elaborate attentional processing of motivationally salient and affectively valenced stimuli^73^ was larger when immediacy was present. We argue that the presence of an immediate reward activated the appetitive motivational system in the brain leading to continued attentional allocation to the stimulus. Larger LPP has been reported in response to liked as compared to disliked food items^74^, and to high-probability compared to low-probability rewards^75^. LPP is sensitive to stimuli that are relevant to biological imperatives, and is associated with more flexible and sustained processes, in contrast to automatic capture of attention during earlier stages^43^.

The results showed that the differences in ERP amplitudes between the immediacy conditions were not predictive of individual differences in the degree of preference reversal. There may be two potential reasons for this^31^. One, stable individual differences in ERPs may have been quite small and, given random trial-to-trial noise, were not detectable. Two, the individual differences in ERPs may have reflected non-functional factors that were not related to the differences in the degree of preference reversal. The latter reason would imply that the observed ERPs reflected a state- rather than a trait-related characteristic.

Further, amplitude analysis of ERPs showed the effect of immediacy in frontocentral and parietal regions for P2, and centroparietal and occipital regions for LPP. Whereas, source localization analysis revealed the effect of immediacy to be primarily centred on the visual cortex in the occipital region during both P2 and LPP. Since there are limitations in inferring distributions of neural generators from distributions of scalp potentials, there have been suggestions to maintain the distinction between both and not to draw strong conclusions from condition x electrode site interactions^31,76,77^. Therefore, in the present study, we relied on the source localization results to infer the neural generators that were affected by immediacy.

The present study also reported two supplementary analyses. In the first supplementary analysis, we categorised the Immediacy Present trials in which the immediate reward was chosen into those that resulted in preference reversal upon the addition of the delay increment and those that did not. The ERPs elicited by these two trial sets were then compared with each other to more directly examine the neural responses implicated in preference reversal. However, due to the low number of trials without preference reversal, data from only a subset of the participants could be included in this analysis. The null findings of this analysis therefore may be due to high noise levels in the data. Future studies should replicate this analysis with more appropriate paradigms that yield adequate (and approximately equal) number of trials with and without preference reversal. In the second supplementary analysis, ERPs elicited by Immediacy Present trials associated with preference reversal were compared with Immediacy Absent trials associated with preference reversal. This was done to more closely examine how immediacy effect resulted in preference reversal by narrowing the focus of analysis to the sub-set of trials that were associated with preference reversal. ERP results of this analysis mirrored the results obtained from the main analysis that compared Immediacy Present condition with Immediacy Absent condition. Source localization did not reveal significant differences after correcting for multiple comparisons during P2 and N2; however, areas of visual cortex had the maximum differential activation, which is consistent with the results of the main analysis. Similarly, during LPP, areas of visual cortex and insula were more active during trials associated with immediacy and preference reversal, signifying that the early activation of insula likely continued also during later parts of the decision making process. The failure to reach statistical significance during P2 and N2 may have been due to increased noise levels in the data due to decreased number of trials. However, overall, the pattern of results of this supplementary analysis agreed with those from the main analysis.

It is noteworthy that the present study extends a body of literature that investigates the neural correlates of evaluating immediate and delayed rewards using ERPs^78–81^. While participants in these previous studies performed valuation of immediate and delayed rewards separately, the present study required participants to process both immediate and delayed rewards in an intertemporal decision context and make preference judgements between them. Although the paradigm used in the present study may not be strictly comparable to these previous studies, their results suggesting over-valuation of and heightened emotional and attentional bias towards immediate rewards are in broad agreement with ours.

There have been several attempts at explaining hyperbolic discounting which allows for immediacy effect and consequent preference reversals to occur. According to a psychophysical argument, the scalar property of time perception (that is, the coefficient of variation of time estimates being a constant) can be shown to elicit hyperbolic discounting of rewards^82,83^, and thus preference reversals. Keren and Roelofsma^16^ put forth the notion that it is the risk that is inherent in delayed rewards that makes them qualitatively different from immediate rewards, which are perceived as certain. Further, as mentioned in the introduction, McClure et al.^24^ argued that an impatient valuation system consisting of limbic structures is preferentially activated when a reward is immediately available, and biases the decision maker towards choosing it.

The present study attempted to shed further light on the question by employing a novel decision making task and using ERPs and source localization. This is the first study to use EEG to investigate immediacy effect and the consequent preference reversal in a decision context. Availability of immediate reward was found to capture both reflexive and sustained attention, and cause significantly more activation in the visual cortex indicating higher valuation. Higher early activation of insula—indicating increased awareness of visceral signals—predicted the degree of impulsive preference reversal, possibly by exerting influence at the selection stage of decision making. To the best of our knowledge, this is the first study reporting a neural correlate predictive of the degree of intertemporal preference reversal. However, the source localization results need to be confirmed in future studies using techniques such as fMRI which have higher spatial resolution. Also, it is important to note that the argument that increased early activation of insula in response to immediate rewards indicates increased awareness of visceral signals is speculative in nature, and future studies should aim to test this explicitly. Availability of immediate reward in the present study was also characterised by diminished engagement of cognitive control processes.

**Figure 11:**
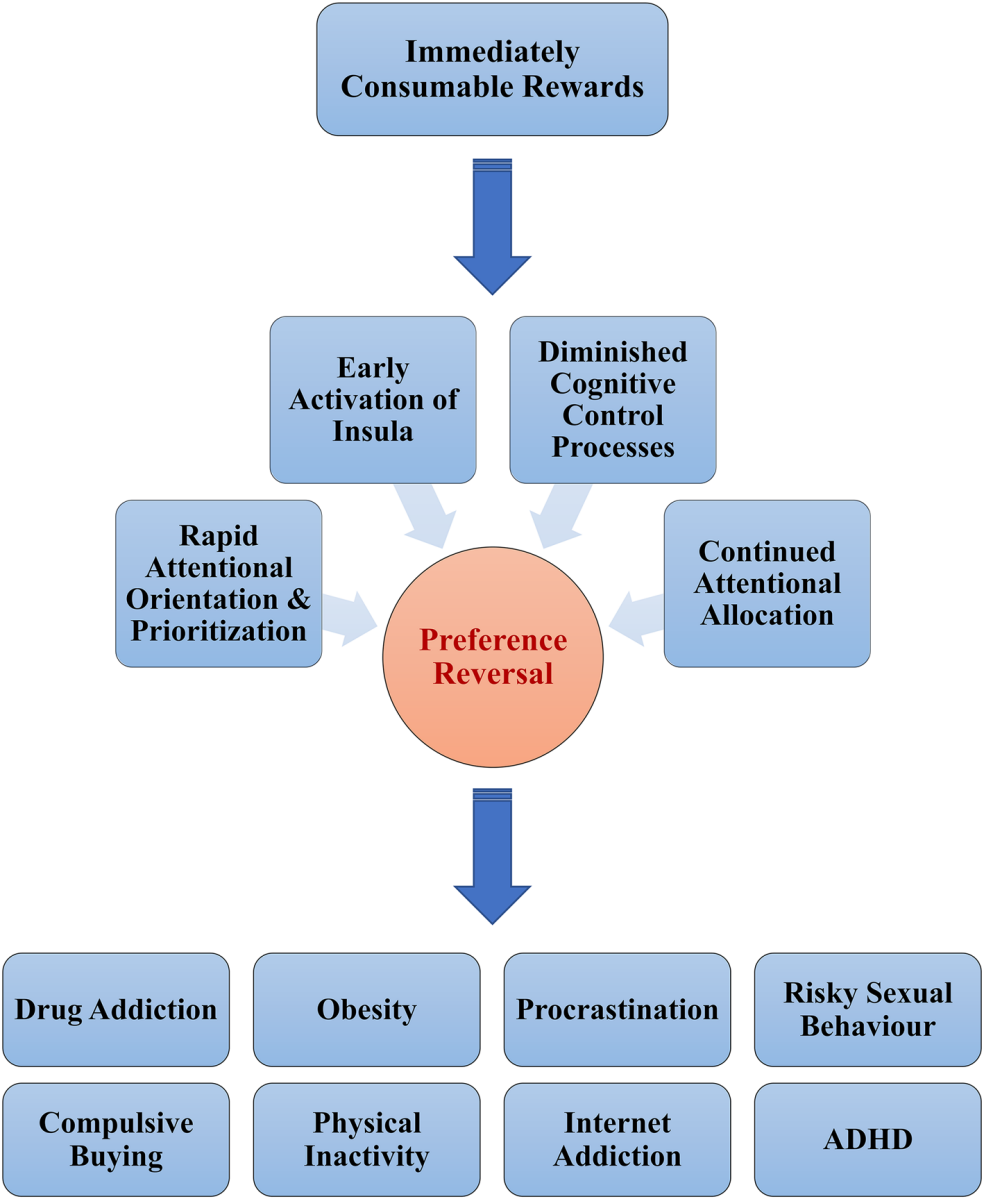
Schematic illustration of the study findings and links to maladaptive behaviours and clinical outcomes. Availability of rewards which are immediately consumable elicits a range of neurocognitive processes which tend to result in reversal of preference from delayed but more beneficial outcomes to immediate ones which are often damaging in the long term. This phenomenon may underlie various impulse control disorders. Note that the link to such disorders is speculative and does not follow directly from the present study.

That insular activation at very early stages of decision making was predictive of behaviour suggests that choices in the presence of immediate rewards in large measure are reflexive, which partly accounts for failures in self-control. These results reinforce the utility of the strategy of precommitment to ensure beneficial outcomes in the long run^7^, which involves eliminating the need to make a choice at the “last minute” by committing to a course of action ahead of time, and thus eliminating the possibility of visceral states precipitated by immediately available rewards biasing decisions. Given that the results also showed immediately available rewards preferentially biased attentional processes due to their high affective and motivational salience, future studies should investigate cognitive strategies that help regulate this process in intertemporal decision contexts. For example, in situations where precommitment is not possible, taking longer duration to decide and using strategies such as deliberately attending to the delayed reward may be helpful in reducing impulsive behaviour. Further, disruptions in neural circuits that balance behaviours that deliver a reward now versus behaviours that give an increased advantage later has been linked to various disorders including addiction, obesity, and attention-deficit/hyperactivity disorder^84^. Over-valuation of immediate rewards within an intertemporal choice context that leads to preference reversals may also underlie maladaptive behaviours such as procrastination, risky sexual behaviour, compulsive buying, and physical inactivity among others (Fig. 11). The present study delineated the neural correlates of such immediacy effect using a novel choice paradigm which may be helpful in understanding these phenomena better. Future studies should build on the present findings by investigating populations with difficulties in delaying gratification and comparing them with healthy individuals to examine the neurocognitive processes that primarily contribute to the emergence of these phenomena.

Finally, it is important to note the limitations of the present study. Firstly, the experiment used hypothetical rewards in the decision making task. Although there is some evidence that hypothetical and real rewards elicit equivalent behavioural^12,46^ and neural^85^ effects in intertemporal choice contexts, this potentially may be a limitation. Secondly, the reward and delay magnitudes were varied only on two levels to examine their effects on preference reversal. Future studies may benefit from varying these over a wider range. Thirdly, a large common delay increment was added to both rewards in the choice task to examine the phenomenon of preference reversal. Future studies should systematically investigate the effect of the magnitude of the common delay increment on the likelihood of preference reversal. Fourthly, the ERPs were locked to the presentation of the smaller-sooner reward which was always presented as the second option. The rationale for doing this was that the experimental manipulation of immediacy was related to the smaller-sooner reward, and since we assumed that the decision process only begins when all information has been presented, this option was presented second. However, this may be a potential confounding factor and should be addressed in future studies using appropriate counterbalancing conditions. Fifthly, the present study contained low number of female participants, and future studies will benefit from collecting data from samples with more balanced distribution of the sexes.

## Methods

### Ethics statement

The study was carried out in accordance with the Declaration of Helsinki and was approved by the Institute Ethics Committee of Indian Institute of Technology Bombay. All participants provided informed consent.

### Participants

Twenty-four participants (3 females) were recruited from a college in Mumbai, India. Participants had a mean age of 23.71 years (*SD* = 2.64 years) and were undergraduate or postgraduate or research scholars in various disciplines. Participants had normal or corrected-to-normal vision and did not have any current medical, psychiatric, or neurological conditions. All participants reported to be right-handed. Participants were instructed to refrain from consuming intoxicating substances 24 h prior to the experiment, and to wash their hair before coming. Participants received a show-up fee of INR 100 (USD 1.40).

### Experimental task

Before beginning the main task, an adapted form of the 27 item Monetary Choice Questionnaire (MCQ) developed by Kirby et al.^86^ was administered to the participant, which asks the participant to make choices between smaller immediate rewards and larger delayed rewards. The purpose of this was to obtain initial estimates of participants’ discount rate which were to be used to construct choice trials in the EEG task. MCQ was adapted by multiplying all reward amounts (both immediate and delayed) by a factor of 10 and expressing them in Indian rupee. This did not alter the basic structure of the questionnaire as the ratio of immediate reward to delayed reward remained same. The amounts were multiplied by 10 as the original amounts were too small when expressed in terms of rupee. The MCQ provided estimates of delay discounting at three levels of reward magnitudes: small (₹ 250–350), medium (₹ 500–600), and large (₹ 750–850), after analysing the responses using the method and syntax described by Gray et al.^87^. The delays used in MCQ ranged from 7 to 186 days. The delay discounting rate for small (*k*_small_) and large (*k*_large_) rewards were used in the subsequent main task.

A novel task was designed to test the effect of immediacy on choices. In the main task during which EEG was recorded, participants had to make a series of choices between smaller-sooner rewards and larger-later rewards. The rewards were hypothetical in nature, but participants were asked to respond as if real money were at stake. To generate the delayed options in the IP condition, we used two levels of reward magnitude (small, large) crossed with two levels of delay magnitude (small, large), where:

- Small delayed reward (*A*_small_) = integer value randomly drawn from [150, 350]
- Large delayed reward (*A*_large_) = integer value randomly drawn from [1100, 1300]
- Small delay (*D*_small_) = integer value randomly drawn from [7, 30]
- Large delay (*D*_large_) = integer value randomly drawn from [180, 210]

This yielded four types of trials: (*A*_small_, *D*_small_), (*A*_small_, *D*_large_), (*A*_large_, *D*_small_), and (*A*_large_, *D*_large_). The reward magnitudes were expressed in Indian rupee (₹) and delays were expressed in days. In the IP condition, the value of the immediate reward, *V,* was derived in each trial as per the 1-parameter hyperbolic delay discounting equation, *V* = *A*/(1 + *kD*), where *A* is the reward that is delayed by *D* days, and *k* is the delay discount rate^10^. The discount rate *k* was updated after every trial based on the response made. If a subject selected the immediate reward in a trial, the value of *k* was increased by 15% in the following trial. Conversely, if a subject selected the delayed reward in a trial, the value of *k* was decreased by 15%. The initial value of *k* was *k*_large_ for trials that used *A*_large_ and *k*_small_ for trials that used *A*_small_. This process ensured that the discount rate used to generate the trials stayed close to the subject’s characteristic discount rate.

The trials generated under IP condition were saved in the computer memory which were used to generate trials under IA condition. This was done by adding a delay *D*_increment_ to both smaller-sooner and larger-later rewards. *D*_increment_ was a random integer value drawn from [290, 310]. Trials under IA and IP conditions were randomly presented in five blocks, with trial type also randomised. After each block, a break of one minute was provided. Forty trials with valid responses were acquired from each trial type in both IP and IA conditions forming a total of 320 trials which were analysed.

Additionally, 20 IA filler trials were used in the beginning of the task so that there were adequate number of IP trials in the memory to start generating and present true IA trials. Towards the end of the task, 20 IP filler trials were used so that the total number of IA and IP trials remained the same. Table 7 shows a summary of the experimental conditions, possible trial types, and the number of trials that were analysed.

**Table 7:** Eight trial types and the ranges of values used to construct trials in the intertemporal choice task.

Immediacy condition	Reward and delay magnitude condition		Range of values	Number of trials analysed
IP	LLR (Rs.)	Small	[150, 350]	40
		Large	[1100, 1300]	40
	LLD (days)	Small	[7, 30]	40
		Large	[180, 210]	40
IA	LLR (Rs.)	Small	[150, 350]	40
		Large	[1100, 1300]	40
	LLD (days)	Small	[7, 30] + [290, 310]	40
		Large	[180, 210] + [290, 310]	40

Smaller-Sooner Reward was computed for each trial using either *k*_small_ or *k*_large_ as per the hyperbolic delay discounting equation (see “Methods”). Smaller-Sooner Delay was 0 for IP condition and [290, 310] for IA condition.

*IP* immediacy present, *IA* immediacy absent, *LLR* larger-later reward, *LLD* larger-later delay.

**Figure 12:**
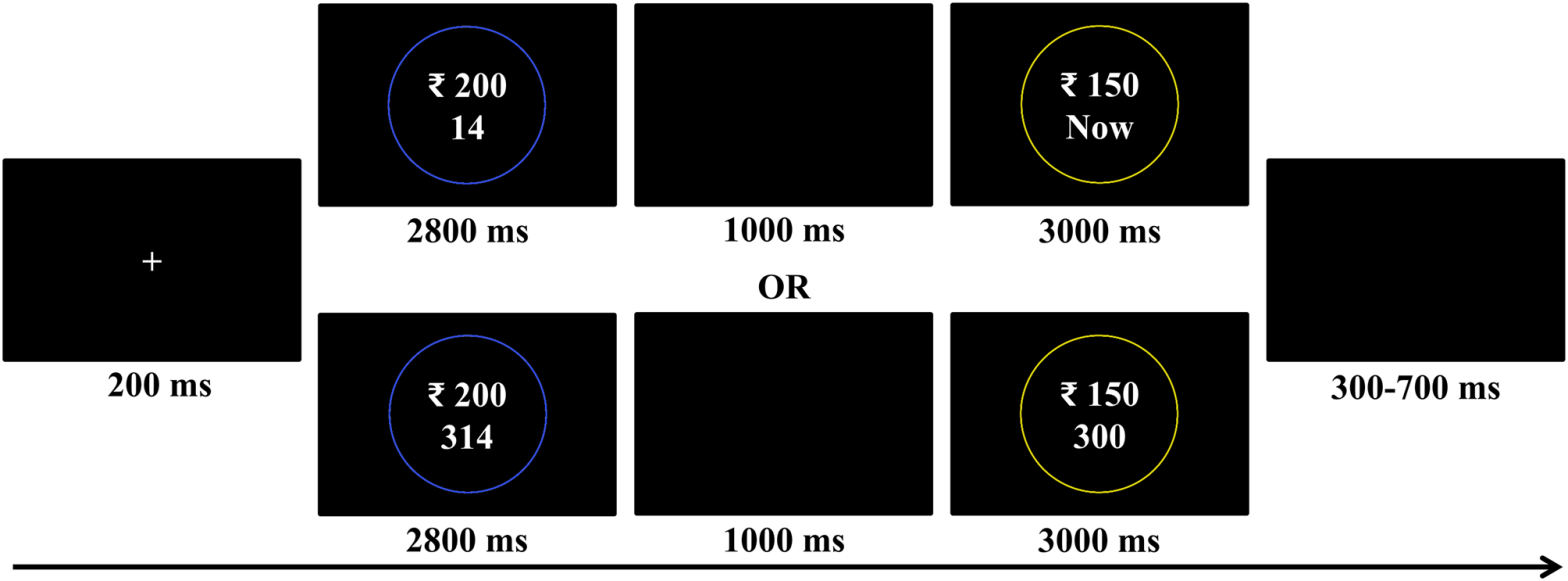
Schematic illustration of a trial. First the larger-later reward was presented, followed by smaller-sooner reward. In Immediacy Present trials, the smaller-sooner reward was immediately available, while in Immediacy Absent trials, both rewards were delayed.

At the beginning of each trial (Fig. 12), a fixation cross was displayed for 200 ms, after which the larger-later reward was shown in a blue/yellow circle for 2800 ms. This was followed by a blank screen for 1000 ms. After this, the smaller-sooner reward was displayed for 3000 ms in a yellow/blue circle, during which the subject had to indicate their preference. In IP trials, the smaller-sooner reward was immediately available, while in IA trials, both rewards were delayed. The association between colour of the circle and the reward was counterbalanced across participants. Subjects had to press the key ‘a’ using left index finger to choose the larger-later reward and ‘6’ using right index finger to choose the smaller-sooner reward. The association between the keys and the choice options too was counterbalanced across participants. If they failed to respond during the time window, the trial was re-done using a fresh set of parameters. Finally, a blank screen was shown for a random duration between 300 and 700 ms before proceeding to the next trial.

### Barratt Impulsiveness Scale

Barratt Impulsiveness Scale (BIS-11)^88^ is a 30 item self-report questionnaire that measures trait impulsivity. Items are scored on a Likert scale (1 = *rarely/never* to 4 = *almost always/always*). The scale had a Cronbach’s alpha reliability of 0.82.

### Apparatus

EEG was recorded using BioSemi Active-Two amplifier system (Biosemi, Amsterdam, the Netherlands), using 64 Ag/AgCl active electrodes mapped according to the 10–20 international system for electrode placement. Two additional electrodes common mode sense (CMS) and driven right leg (DRL) were used as the reference and the ground respectively. E-Prime 2.0 (Psychology Software Tools Inc. Pittsburgh, PA, USA) was used to create and present the experimental stimuli and to record participants’ responses. The display screen had a resolution of 1920 × 1080 and a refresh rate of 60 Hz.

### EEG data acquisition and processing

The electrodes were mounted using an elastic cap and electrolyte gel was used to ensure connectivity to the scalp. Electrode impedances were maintained below 10 kΩ. Signals were sampled at 2048 Hz using an online 0.16 Hz high-pass and 100 Hz low-pass filter. ActiView (BioSemi) software was used for signal acquisition. Triggers marking different events during the course of a trial were communicated from the stimulus presentation computer to the ActiView software via parallel port.

Brain Electrical Source Analysis 6.0 (BESA; Gräfelfing, Germany) was used for offline pre-processing and averaging the data. Eye-blink artifacts were corrected using a semi-automatic method by identifying them through a template-based method and by modelling artifact topography^89^. The signals were re-referenced to the average of the mastoids. Epochs were defined as − 200 ms to 1000 ms relative to the presentation of the smaller-sooner option. Baseline correction was performed based on the pre-stimulus interval of 200 ms. Epochs with signal amplitudes exceeding ± 100 µV were excluded from averaging. Noisy EEG data was excluded also through visual inspection. The average waveforms were then computed for IP and IA conditions. The mean percentage of trials utilised for averaging were 86.00 ± 6.16 and 84.75 ± 7.13 for IP and IA conditions respectively, with no difference across the conditions (*t*(23) = 1.32, *p* = 0.20). The average waveforms were low-pass filtered at 30 Hz. Relevant literature along with a collapsed localizer approach were utilised to determine the electrodes and time windows for ERP analyses^31,90^. This approach involves averaging the waveforms across the conditions and then identifying temporal windows and set of electrodes to be used for the non-collapsed data, thus avoiding bias towards any specific condition. P2 was analysed at the frontocentral (Fz, F3, F4, FCz, FC3, FC4, Cz, C3, and C4), parietal (Pz, P3, P4, CPz, CP3, and CP4)^35^, and occipital sites (POz, PO3, PO4, Oz, O1, and O2) in the time window 200–300 ms. N2 was analysed at the frontal sites (Fz, F3, F4, FCz, FC3, and FC4)^32^ in the time window 300–370 ms. LPP was analysed at the centroparietal (Cz, C1, C2, CPz, CP1, CP2, Pz, P1, P2) and occipital (POz, PO3, PO4, Oz, O1, O2) sites^43^ in the time window 400–900 ms.

We also conducted two supplementary analyses. In the first supplementary analysis, in order to more directly examine the neural responses that underlay preference reversal, IP trials in which immediate reward was chosen were classified into two sets for each subject: those that resulted in preference reversal upon the addition of the delay increment and those that did not. The mean number of trials in the preference reversal (IP-PR) set was 67.96 ± 33.42, whereas the same in the no preference reversal (IP-NPR) set was 30.33 ± 22.49. Data from only those subjects who had at least 20 artifact-free trials in both the sets of trials were used for this analysis. The cut-off was chosen as there is some evidence for 20 trials being the minimum requirement for obtaining reliable ERP forms that are relevant for the present study^91–93^. Only 10 subjects qualified this criterion. Epochs were defined as − 200 ms to 1000 ms relative to the presentation of the immediate reward, with baseline correction done based on the pre-stimulus interval of 200 ms. Noisy EEG data was excluded in the same manner as described earlier in this section and average waveforms were computed for PR and NPR sets of trials. The mean number of trials used for averaging were 54.40 ± 25.46 and 34.00 ± 8.65 for IP-PR and IP-NPR sets respectively. Although the two sets differed on the number of trials (*t*(9) = 2.32, *p* = 0.04), mean amplitude as the dependent measure has been argued to be unaffected by this asymmetry^31^. The average waveforms were low-pass filtered at 30 Hz. The ERPs (P2, N2, and LPP) were analysed at the same sites and time windows as described earlier in this section.

In the second supplementary analysis, IP trials with immediate choice that resulted in preference reversal upon the addition of the delay increment (IP-PR) were contrasted against the corresponding IA trials (IA-PR; that is, IA trials with delayed choice, each of which had an IP counterpart with immediate choice). This was done to more closely examine the neural correlates of immediacy effect leading to preference reversal by narrowing the analysis only to the sub-set of trials that were associated with preference reversal. The mean number of trials in IP-PR and IA-PR sets was 67.96 ± 33.42 each. Data from two subjects were removed as they had less than 20 trials. Epochs were defined as − 200 ms to 1000 ms relative to the presentation of the smaller-sooner option, with baseline correction done based on the pre-stimulus interval of 200 ms. Noisy EEG data was excluded and average waveforms were computed for IP-PR and IA-PR sets of trials. The mean number of trials used for averaging were 59.14 ± 25.20 and 59.41 ± 24.09 for IP-PR and IA-PR sets respectively, with no difference across them (*t*(21) = − 0.29, *p* = 0.78). The averaged waveforms were low-pass filtered at 30 Hz and the ERPs (P2, N2, and LPP) were analysed at the same sites and time windows as described earlier.

### Source localization

Standardized low-resolution brain electromagnetic tomography (sLORETA) was used for source localization of ERP components that were sensitive to immediacy effect. sLORETA is a method that provides a solution to the inverse problem and it estimates the localization of brain function by computing cortical three-dimensional distribution of current density from electric potential distribution measured on the scalp^94,95^. Computations were done in a realistic head model^96^ using the MNI152 template^97^. The solution space was restricted to cortical grey matter, as determined by the probabilistic Talairach atlas^98^, and consisted of 6239 voxels sized 5 cubic mm. Areas of activation are reported in terms of MNI (Montreal Neurological Institute) coordinates and Brodmann Areas (BAs). sLORETA has been shown to have no localisation bias under realistic conditions^99^, and has been validated through simultaneous EEG/fMRI studies^100,101^.

Functional sLORETA images were computed for IP and IA conditions for each subject. The difference in cortical activity between the conditions (IP minus IA) in the duration of the selected ERP components were assessed using Statistical non-Parametric Mapping (SnPM) with 5000 permutations implemented in the sLORETA program package^95^. SnPM uses a non-parametric randomization procedure that applies a correction of significance for multiple testing and does not require the assumption of Gaussianity^94,102^. Voxel-by-voxel within-group comparisons of the current density distribution were performed for all time frames by employing *t*-statistic on log transformed data. This analysis generated maps of *t*-statistic for each voxel and associated corrected *p* values, enabling the identification of cortical sources that differed significantly between conditions (corrected *p* < 0.05). Correlational analysis between individual differences in the degree of preference reversal (computed by subtracting the percentage of impulsive choices in IA condition from the percentage of impulsive choices in IP condition for each subject) and the differential cortical activations (computed by subtracting sLORETA images of IA condition from sLORETA images of IP condition for each subject) at the whole brain level was also performed. This analysis also utilised SnPM with 5000 permutations which applied a correction of significance for multiple testing. As an extension of the second supplementary analysis, the difference in cortical activity between IP-PR and IA-PR sets of trials was also assessed.

### Statistical analysis

Occurrence of preference reversal due to immediacy was evaluated along with the effects of reward and delay magnitude using repeated-measures analysis of variance (ANOVA). Immediacy (Immediacy Present, Immediacy Absent), Reward Magnitude (Small, Large), and Delay Magnitude (Small, Large) were defined as within-subjects factors, and percentage of impulsive choices was used as the dependent variable. As there were no discernible effects of reward and delay magnitude on choice, data were collapsed across the levels of each of these variables for subsequent analyses of the neurophysiological data to improve statistical power and to reduce false positives. ERP data were analysed using repeated-measures ANOVAs with Immediacy (IA vs IP) and Region/Electrode Position as within-subjects factors and mean amplitude of the averaged waveform as the dependent variable. Repeated-measures ANOVAs with Preference Reversal Occurrence (IP-PR vs IP-NPR) and Region/Electrode Position as within-subjects factors and mean amplitude of the averaged waveform as the dependent variable were also performed. In addition, repeated-measures ANOVAs with Immediacy-PR (IP-PR vs IA-PR) and Region/Electrode Position as within-subjects factors and mean amplitude of the averaged waveform as the dependent variable were conducted. For all ANOVAs, Greenhouse–Geisser corrections were applied whenever sphericity could not be assumed and Bonferroni corrections were used for post-hoc pairwise comparisons. Correlational analyses were conducted to estimate the relationships between scores on BIS-11 and degree of preference reversal, and between the differences in ERP amplitudes between the immediacy conditions and degree of preference reversal.

### Procedure

Prior to the experimental task, participants provided written informed consent. They were seated in a recliner chair in a dimly lit, sound damped room with their hands comfortably resting on a keyboard. Participants filled the Monetary Choice Questionnaire after which they were given instructions on the experimental procedure. They were fitted with the elastic EEG cap and electrodes were attached to it after applying the electrolyte gel. They were given 20 training trials before the main task began. After the experiment they filled Barratt Impulsiveness Scale. Participants were thanked, debriefed, and compensated at the end of the session.

## Data Availability

The datasets generated during and/or analysed during the current study are available from the corresponding author on reasonable request.
